# Clinical Outcomes Comparison of Combined Small Incision Lenticule Extraction with Collagen Cross-Linking Versus Small Incision Lenticule Extraction Only

**DOI:** 10.1155/2022/2625517

**Published:** 2022-10-11

**Authors:** Ayoub Chabib, Massimo Mammone, Chiara Fantozzi, Rebecca R. Lian, Natalie A. Afshari, Michael H. Goldbaum, Marco Fantozzi

**Affiliations:** ^1^Department of Ophthalmology, Faculty of Medicine, “La Sapienza” University of Rome, Rome, Italy; ^2^Department of Ophthalmology Cornea and Refractive Surgery, San Rossore Medical Center, Pisa, Italy; ^3^Department of Ophthalmology, Catholic University, Policlinico Gemelli Hospital, Rome, Italy; ^4^Department of Ophthalmology Cornea and Refractive Surgery, University of California San Diego, La Jolla, CA, USA

## Abstract

**Purpose:**

To evaluate clinical outcome during 24 months follow-up between small incision lenticule extraction combined with cross-linking (SMILE Xtra) and small incision lenticule extraction (SMILE) only. *Setting*. Ophthalmology Division of San Rossore Medical Center, Pisa, Italy.

**Design:**

Retrospective comparative case series.

**Methods:**

The study comprised 70 eyes (35 patients); 40 eyes were corrected using SMILE and 30 eyes were corrected using SMILE Xtra using a low energy protocol. The outcomes were compared at 1, 6, 12, and 24 months postoperatively.

**Results:**

The mean spherical equivalent (SEQ) reduced from −7.18 ± 1.21 D to −0.01 ± 0.09 D in the SMILE group and from −6.20 ± 2.99 D to −0.04 ± 0.1 D postoperatively in SMILE Xtra (*p* < 0.05). At 24 months the mean SEQs were −0.01 ± 0.24 D for SMILE and −0.15 ± 0.33 D for SMILE Xtra (*p* > 0.05). At 1, 6, 12, and 24 months, there were no statistically significant differences between the SMILE and SMILE Xtra groups in logarithm of the minimum angle of resolution (logMAR) uncorrected distance visual acuity (UDVA), safety, and efficacy index (*p* > 0.05). The mean average keratometry (*K*-avg) at 1, 6, 12, and 24 months after surgery did not shown any statistically significant difference between SMILE and SMILE Xtra group (*p* > 0.05). The mean maximum keratometry (*K*-max) readings at 1, 6, 12, and 24 months were not statistically significant between SMILE and SMILE Xtra group (*p* > 0.05). The preoperative mean thinnest point pachymetry (TTP) was 543.90 ± 22.85 *μ*m in the SMILE group and 523.40 ± 37.01 *μ*m in the SMILE Xtra group (*p* < 0.05). At 1, 6, 12, and 24 months the mean TTP was not statistically significant between the SMILE and SMILE Xtra groups (*p* > 0.05). At 24 months, the TTP was 408.29 ± 38.75 *μ*m for the SMILE group and 402.22 ± 37 *μ*m for the SMILE Xtra group (*p* > 0.05). In the preoperative period, the mean maximum posterior elevation (MPE) was 8.63 ± 4.35 *μ*m for SMILE and 8.13 ± 2.54 *μ*m for SMILE Xtra (*p* > 0.05). After the surgical procedure, both groups showed a statistically significant increase of the MPE (*p* < 0.05). At 24 months, the MPE was 11.00 ± 4.72 *μ*m for SMILE Xtra and 10.14 ± 3.85 *μ*m for the SMILE group (*p* > 0.05). In the preoperative period, the means of the root mean square (RMS) of high-order aberration (HOA) were 0.08 ± 0.03 *μ*m for the SMILE group and 0.08 ± 0.03 *μ*m for the SMILE Xtra group (*p* > 0.05). At 24 months, the RMS of HOA was 0.13 ± 0.07 *μ*m for the SMILE group and 0.14 ± 0.07 *μ*m for the SMILE Xtra group (*p* > 0.05). In the preoperative period, the root mean square of coma aberration (RMS-Coma) aberration was 0.06 ± 0.09 *μ*m for the SMILE group and 0.04 ± 0.03 *μ*m for the SMILE Xtra group (*p* > 0.05). At 24 months, the coma aberration of SMILE group was 0.12 ± 0.21 *μ*m and 0.16 ± 0.25 *μ*m for SMILE Xtra group (*p* > 0.05).

**Conclusions:**

SMILE Xtra procedure is a safe and simple procedure that can be offered to patients with high corneal ectasia risk because there were no differences in the indices of ectasia compared to the group treated only with SMILE which has a low corneal ectatic risk.

## 1. Introduction

The decision for refractive surgery in patients with high myopia, borderline topography, and thinner corneas is challenging. One of the most feared complications is postoperative ectasia, a progressive steepening, and thinning of the cornea [1–4].

Collagen cross-linking (CXL) has been proven to be an effective modality to strengthen and stabilize the cornea in keratoconus and ectasia after corneal refractive surgery [5–8]. CXL has also been used with good results in combination with photoablative procedures, such as laser assisted in situ keratomileusis (LASIK) and photorefractive keratectomy (PRK), to improve the safety profile in patients who had risk factors for corneal ectasia [9, 10].

The small incision lenticule extraction (SMILE) technique provides a biomechanically stronger cornea since it preserves the integrity of the anterior cornea. Although a large case series has demonstrated an excellent safety profile for SMILE [11], corneal ectasia after SMILE in eyes with thinner corneas and borderline topography has been reported [12, 13]. Refractive lenticular extraction (ReLEx) SMILE, when combined with CXL intraoperatively, may further prevent the risk of future ectasia in susceptible individuals.

The purpose of this study is to assess the 2-year clinical outcome of a series of small incision lenticule extraction combined with cross-linking (SMILE Xtra) cases using a low-energy protocol compared to SMILE alone.

## 2. Materials and Methods

### 2.1. Study Design

This study was a retrospective interventional comparative study. The patients of the current study signed an informed consent before the intervention. The study was approved by the Institutional Ethics Committee and adhered to the tenets of the Declaration of Helsinki. The data were anonymized or maintained with confidentiality.

### 2.2. Study Population

The first group included 30 eyes of 15 patients who underwent SMILE Xtra procedure. The second control group included 40 eyes of 20 patients, who were treated with the SMILE procedure only. Inclusion criteria of the study included moderate to high myopia with baseline spherical equivalent (SEQ) higher than 4 diopters, and all eyes had a emmetropic target refraction.

Exclusion criteria included subjects with corneal thickness <450 *μ*m, established keratoconus, family history of keratoconus or eye rubbing [14], hyperopic refractive error, mixed astigmatism, concurrent eye infection, history of riboflavin allergy, past history of herpes infection or chemical injury, long-term topical or oral steroid use, and pregnant or nursing females.

All included patients had preoperative complete ophthalmic clinical examination including slit-lamp examination, intraocular pressure measurement, fundus examination, dry eye assessment, cycloplegic refraction, uncorrected distance visual acuity (UDVA), and corrected distance visual acuity (CDVA) measured with Snellen chart at 6 m, which was converted to minimum angle of resolution (logMAR) value. According to the Randleman Scoring for ectasia risk, eyes were classified into low (score ≤2), and moderate to high risk (score ≥3) [15]. The SMILE Xtra procedure was offered to patients with a moderate to high ectasia risk score while SMILE treatment was offered to patients with low risk [16]. The SMILE Xtra group has a higher score than the SMILE group despite having similar characteristics, with the exception of corneal thickness and topographic morphology. Although this scoring system was developed for LASIK, it was applied here because a modified version does not yet exist for SMILE.

### 2.3. Surgical Techniques

A single surgeon performed all the surgeries (M.F.) using an established technique under topical anesthesia. The SMILE procedure was performed using the 500 kHz VisuMax femtosecond laser (Carl Zeiss Meditec). The following parameters were used: cap thickness 130–140 *μ*m; cap diameter 7.0–7.5 mm; lenticule diameter 6.0–6.5 mm with a transition zone of 0.1 mm; cut energy 1.4 J; spot and tracking distance 2.0–3.0 *μ*m. A 2 mm incision located at 10 o'clock position for both eyes was performed. A blunt spatula was used to separate the lenticule, which was then removed by forceps through the incision. Finally, the corneal interface was flushed with balanced salt solution. In SMILE Xtra cases, after lenticule removal, 0.22% riboflavin with saline (VibeX Xtra, Avedro) was injected through the small incision into the interface and left to soak for 90 seconds, followed by UV-A irradiation at 30 mW/cm2 for 90 seconds (total energy: 2.7 J/cm^2^) using the Avedro KXL system.

Postoperative medications included ophthalmic topical tobramycin 0.3% and dexamethasone 0.1% suspension four times a day for 1 week and then steroids eye drops were tapered over 1 month. Finally preservative-free artificial teardrops were given for 3 months postoperatively. All patients were examined preoperatively and postoperatively at day 1, 1 week, 1, 6, 12, and 24 months.

### 2.4. Outcome Measurements

The visual acuity (Snellen chart at 6 m) was converted to logMAR value, and refraction was recorded at 1, 6, 12, and 24 months. The corneal tomography (MS-39; CSO), corneal topography (Sirius; CSO) and aberrometry (Osiris; CSO) were performed. The following data were recorded: average keratometry (*K*-avg), maximum keratometry (*K*-max), corneal astigmatism, thinnest point pachymetry (TTP), mean maximum posterior elevation (MPE), means of the root mean square of high-order aberration (RMS-HOA), and root mean square of coma aberration (RMS-Coma). The efficacy index was calculated as the ratio of postoperative UDVA to preoperative CDVA, and the safety index was determined as the ratio of postoperative CDVA to preoperative CDVA.

### 2.5. Statistical Analysis

Statistical analysis was performed using the SPSS software for Mac (IBM, Version 20.0.0). The parameters between SMILE Xtra and control group were compared with MannWhitney *U* test for age, Fisher's exact test for gender, and linear mixed model analysis for other parameters. The difference was considered statistically significant when the associated *p* value was less than 0.05. All values were expressed as mean ± SD.

## 3. Results

A total of 40 eyes for the SMILE group and 30 eyes for the SMILE Xtra group was included for analysis. The baseline demographic and preoperative data are summarized in Table 1. Both groups had comparable age, gender distribution, and SEQ (*p* > 0.05). According to the Randleman Scoring, 30 SMILE Xtra eyes (100%) had an ectasia risk factor score of 3 or higher (moderate/high risk). In the control group, 40 eyes (100%) had a score of 2 or lower (low risk). No intraoperative or postoperative complications occurred in either group. Postoperatively, there was no clinically detectable corneal haze in all cases.

### 3.1. Refraction, Visual Acuity, Safety, and Efficacy

**Figure 1:**
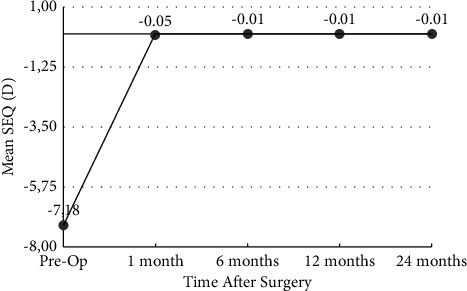
Stability of manifest refraction spherical equivalent over time in the SMILE group (*y*-axis: spherical equivalent in diopters; *x*-axis: postoperative time interval in months).

**Figure 2:**
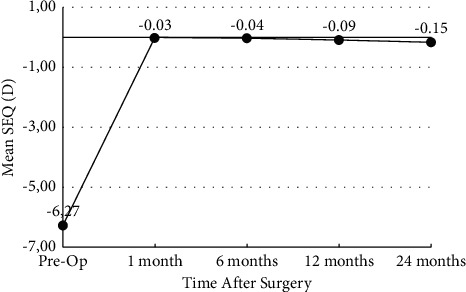
Stability of manifest refraction spherical equivalent over time in SMILE Xtra group (*y*-axis: spherical equivalent in diopters; *x*-axis: postoperative time interval in months).

The mean SEQ reduced from −7.18 ± 1.21 D to −0.05 ± 0.11 D in the SMILE group and from −6.27 ± 2.95 D to −0.03 ± 0.08 D at 1 month postoperatively in SMILE Xtra (*p* < 0.05). At 12 months, the mean errors in the correction in the SEQ were −0.01 ± 0.21 D for SMILE and −0.09 ± 0.18 D for SMILE Xtra (*p* > 0.05). The mean errors in the correction in the SEQ at 24 months were −0.01 ± 0.24 D for SMILE and −0.15 ± 0.33 D for SMILE Xtra (*p* > 0.05) (Figures 1 and 2).

**Figure 3:**
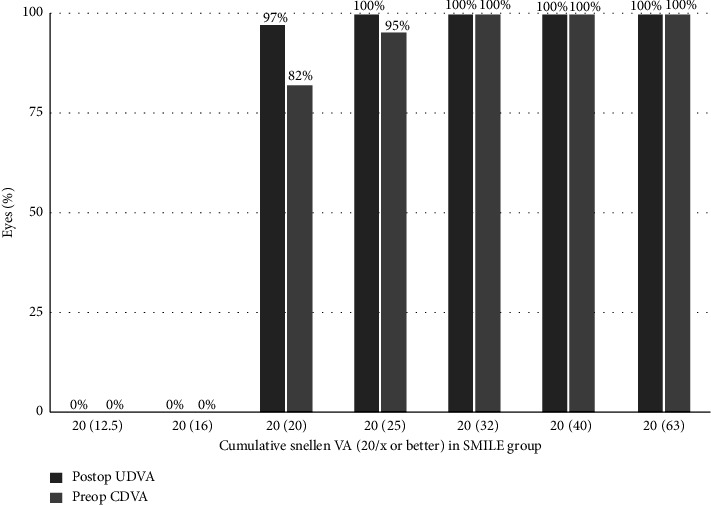
Cumulative Snellen visual acuity in SMILE group: preop CDVA and post UDVA.

**Figure 4:**
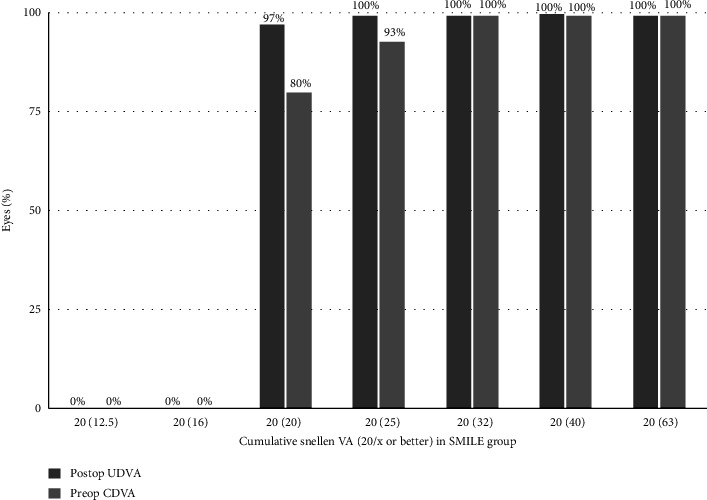
Cumulative Snellen visual acuity in SMILE Xtra group: preop CDVA and post UDVA.

The UDVA remained stable over time in both groups, with no eye losing more than 1 line of visual acuity. At 12 months, in the SMILE group, 38 eyes (95%) achieved UDVA 20/20 and only 2 eyes had UDVA 20/25. In the SMILE Xtra group, 29 eyes (96.66%) achieved UDVA 20/20 and only 1 eye had UDVA 20/25. At 24 months, in the SMILE group, 39 eyes (97.5%) achieved UDVA 20/20 and only 1 eye had UDVA 20/25. In the SMILE Xtra group, 29 eyes (96.66%) achieved UDVA 20/20 and only 1 eye had UDVA 20/25 (Figures 3 and 4).

There was no statistically significant difference in logMAR UDVA at 1, 6, 12, and 24 months after surgery between the SMILE and SMILE Xtra groups (*p* > 0.05).

The safety index and efficacy index of both groups at 1, 6, 12, and 24 months are summarized in Table 2. The difference between both indices was not statistically significant during all follow-up between the SMILE and SMILE Xtra groups (*p* > 0.05).

### 3.2. Keratometry Values

Preoperatively, there was no significant difference in *K*-avg values among SMILE and SMILE Xtra group (43.86 ± 1.34 D vs. 44.13 ± 1.18 D; *p* > 0.05) (Table 1). The *K*-avg values decreased following surgical procedures for both groups (*p* < 0.05). At month 24 after surgery, no statistically significant difference in *K* values between the two groups (38.74 ± 1.76 D vs. 38.35 ± 1.85 D; *p* > 0.05) was observed.

As shown in Table 3, the *K*-avg at month 1, 6, 12, and 24 months after surgery, did not show any statistically significant difference between the SMILE and SMILE Xtra groups (*p* > 0.05).

The *K*-max values difference was not statistically significant in preoperative time between the SMILE and SMILE Xtra groups. After the surgical procedure, both groups showed a statistically significative increase of *K*-max (*p* < 0.05). The *K*-max readings at 1, 6, 12, and 24 months were not statistically significant between the SMILE and SMILE Xtra groups (*p* > 0.05) (Table 3).

### 3.3. Thinnest Point Pachymetry

**Figure 5:**
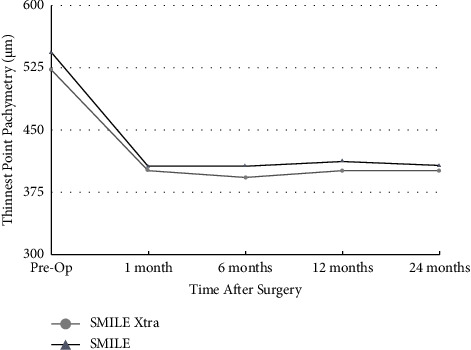
Thinnest point pachymetry following SMILE and SMILE Xtra.

The means of the postoperative TPP were 543.90 ± 22.85 *μ*m in the SMILE group and 523.40 ± 37.01 *μ*m in the SMILE Xtra group (*p* < 0.05) (Table 1). After the surgical procedure, both groups showed a statistically significative reduction of TPP (*p* < 0.05). At 1 month, the TPP means were 403.83 ± 35.51 *μ*m for the SMILE group and 399.40 ± 40.82 *μ*m for the SMILE Xtra group (*p* > 0.05). At 6 months, the TPP means were 407.94 ± 37.05 *μ*m for the SMILE group and 394.45 ± 38.00 *μ*m for the SMILE Xtra group (*p* > 0.05). At 12 months, the TPP means were 414.03 ± 36.28 *μ*m for the SMILE group and 402.16 ± 39.52 *μ*m for the SMILE Xtra group (*p* > 0.05). At 24 months, the TPP means were 408.29 ± 38.75 *μ*m for the SMILE group and 402.22 ± 37 *μ*m for SMILE Xtra (*p* > 0.05) (Table 3, Figure 5).

### 3.4. Maximum Posterior Elevation

Preoperatively, the MPE means were 8.63 ± 4.35 *μ*m for SMILE and 8.13 ± 2.54 *μ*m for SMILE Xtra (*p* > 0.05) (Table 1). After the surgical procedure, both groups showed a statistically significant increase of the MPE (*p* < 0.05). At 1 month, the MPE means were 13.22 ± 5.86 *μ*m for the SMILE Xtra group and 12.65 ± 5.85 *μ*m for the SMILE group (*p* > 0.05). At 6 months, the MPE means were 14.81 ± 7.02 *μ*m for the SMILE Xtra group and 12.47 ± 7.33 *μ*m for the SMILE group (*p* > 0.05). At 12 months, the MPE means were 11.54 ± 5.15 *μ*m for the SMILE Xtra group and 11.13 ± 4.39 *μ*m for the SMILE group (*p* > 0.05). At 24 months, the MPE means were 11.00 ± 4.72 *μ*m for the SMILE Xtra group and 10.14 ± 3.85 *μ*m for the SMILE group (*p* > 0.05) (Table 3).

### 3.5. High-Order Aberration

Preoperatively, the mean RMS-HOA was 0.08 ± 0.03 *μ*m for the SMILE group and 0.08 ± 0.03 *μ*m for the SMILE Xtra group (*p* > 0.05) (Table 1). After the surgical procedure, both groups showed a statistically significant increase of HOA (*p* < 0.05). At 1 month, there was no statistically significant difference between the SMILE (0.17 ± 0.10 *μ*m) and SMILE Xtra groups (0.17 ± 0.09 *μ*m) (*p* > 0.05). At 6 months, the mean RMS-HOA was 0.20 ± 0.20 *μ*m for the SMILE group and 0.19 ± 0.14 *μ*m for the SMILE Xtra group (*p* > 0.05). At the 12-month follow-up, the mean RMS-HOA was 14 ± 0.07 *μ*m for the SMILE group and 0.12 ± 0.06 *μ*m for the SMILE Xtra group (*p* > 0.05). At 24 months, the mean RMS-HOA was 0.13 ± 0.07 *μ*m for the SMILE group and 0.14 ± 0.07 *μ*m for the SMILE Xtra group (*p* > 0.05) (Table 4).

### 3.6. Coma Aberration

Preoperatively, the mean RMS-Coma aberration was 0.06 ± 0.09 *μ*m for SMILE and 0.04 ± 0.03 *μ*m for SMILE Xtra (*p* > 0.05) (Table 1). After the surgical procedure, both groups showed a statistically significant increase of the coma aberration (*p* > 0.05). At 1 month, the mean RMS-Coma aberration was 0.12 ± 0.15 *μ*m for SMILE and 0.16 ± 0.18 *μ*m for SMILE Xtra (*p* > 0.05). At 6 months, the mean RMS-Coma aberration was 0.14 ± 0.34 *μ*m for the SMILE group and 0.08 ± 0.10 *μ*m for the SMILE Xtra group (*p* > 0.05). At 12 months, the mean RMS-Coma aberration was 0.06 ± 0.07 *μ*m for the SMILE group and 0.06 ± 0.05 *μ*m for the SMILE Xtra group (*p* > 0.05). At 24 months, the mean RMS-Coma aberration of SMILE was 0.12 ± 0.21 *μ*m and 0.16 ± 0.25 *μ*m for the SMILE Xtra group (*p* > 0.05) (Table 4).

## 4. Discussion

SMILE is a flapless procedure with the theoretical advantage of producing less weakening of the cornea, since the cap thickness contributes to the residual stromal bed. For this reason, SMILE is better option in treating higher myopic errors, thinner corneas, and cases with abnormal topography or *forme-fruste* keratoconus [17, 18]. Despite this advantage, there are reports of corneal ectasia after SMILE [12, 19–21].

Any corneal laser refractive procedure would certainly decrease the biomechanical stability of the cornea. Combining corneal collagen cross-linking with PRK or LASIK showed good results and has come into practice [9, 22]. In 2009, Kanellopoulos published a study demonstrating that a femtosecond laser to create a corneal pocket can be performed as an alternative procedure to the conventional CXL. The procedure has advantages of not removing the epithelium, thus promoting faster healing, better comfort, and lower chances of infection [23]. Based on this idea, the SMILE Xtra procedure was developed, combining corneal collagen cross-linking with the SMILE procedure. Several publications show that the SMILE Xtra is a safe and effective procedure in the short-term period, but there is a lack of long-term safety data [18, 24–26].

The aim of SMILE Xtra is to deliver the least amount of energy that can stabilize the cornea. Excess energy may cause haze while too little energy may be insufficient to ensure corneal strength. Whereas established CXL protocols for treating keratoconus with long-term follow-up have been shown to be safe and effective [27], there are no standardized procedures for prophylactic use of CXL in refractive surgery. In the laser assisted in situ keratomileusis combined with cross-linking (LASIK Xtra) procedure, various authors have used 30 mW/cm^2^ for different durations, with a total energy of 1.8 to 5.4 J/cm^2^, and all those different regimens proved to be safe and effective [10, 18, 22].

In the preoperative time, the SMILE Xtra eyes had a significantly thinner TPP than the control and topographic alteration with an increased risk of ectasia. Our CXL protocol for the SMILE Xtra procedure involves the application of riboflavin directly to the stromal pocket after lenticule extraction followed by UV-A irradiation with a total energy of 2.7 J/cm^2^ (30 mW/cm^2^ for 90 seconds). This amount of energy proved to be safe and well tolerated [28], as none of the cases suffered from haze or any other serious complication, like epithelial defects, deep lamellar keratitis, or punctate keratitis. Other authors have proposed several protocols. Ganesh and Brar reported good outcomes at 12 months for accelerated cross-linking with UV-A radiation at 365 nm wavelength, with 45 mW/cm^2^ of energy delivered for 75 seconds (total energy: 3.4 J/cm^2^) [18]. Ng et al. proposed a different protocol to deliver UV-A at 18 mW/cm^2^ for 45 seconds (total energy: 0.8 J/cm^2^) [24]. Graue-Hernandez et al. reported good refractive outcomes at 24 months for SMILE Xtra by applying the standard Dresden protocol with 5.4 J/cm^2^ total energy [25].

In our study, both SMILE and SMILE Xtra results showed excellent refractive outcomes. The UDVA were comparable between two groups, with refractive predictability reported in the literature [24, 29, 30]. Efficacy and safety indices reported stable results along the 24 months of follow-up. In our SMILE cases, the safety index at 6, 12, and 24 months was, respectively, 1.11, 1.03, and 1.07. This was comparable to the series reported by Ivarsen et al., where their safety index at 3 months was 1.05. In SMILE Xtra group, the safety index at 6, 12, and 24 months was, respectively, 1.09, 1.07, and 1.03. No eyes in our series lost more than 1 line of UDVA, similar to SMILE Xtra series reported by Ganesh and Ng [18, 24].

Regarding keratometry, the SMILE Xtra group showed stable *K*-avg and *K*-max values, with no statistically significant difference from the SMILE group. Before the surgical procedure the TPP between the SMILE and SMILE Xtra showed a statistically significant difference. The SMILE Xtra group showed stable TPP from the 6th to 24th month of follow-up. The difference between the study and control group is not statistically significative at 24 months. Also, the MPE showed stable results during the 24 months of follow-up, with no statistically significant difference with SMILE group. The SMILE Xtra aberrometric data demonstrate no statistically significant differences with SMILE group for both RMS-HOA and RMS-Coma aberration.

The advantages of the current study are the comparative nature with a control group of SMILE cases and a follow-up period of 24 months. The limitations of our study are the retrospective nature of the study, the requirement of more cases, and need for a longer duration of follow-up to record the long-term effects. Moreover, it was not possible, until now, to evaluate the surgical techniques SMILE vs SMILE Xtra composed of only high-risk subjects in both groups, in order to reduce confounding variables.

In conclusion, SMILE Xtra procedure is a safe and effective procedure that can be offered to patients undergoing SMILE with corneal ectasia risk.

## Figures and Tables

**Table 1:** SEQ: spherical equivalent; CDVA: corrected distance visual acuity; *K*-avg: Keratometry average; *K*-max: Keratometry maximum; TPP: thinnest point pachymetry; maximum posterior elevation; HOA-RMS: high-order aberration root mean square; Coma-RMS: coma root mean square.

	SMILE	SMILE xtra	*p* value
Male: female	5 : 9	4 : 11	0.187
Age (years)	36.45 ± 8.84	33.20 ± 7.98	0.056
SEQ (D)	−7.18 ± 1.21	−6.27 ± 2.95	0.208
CDVA (logMAR)	−0.02 ± 0.05	−0.03 ± 0.06	0.805
*K*-avg (D)	43.86 ± 1.34	44.13 ± 1.18	0.394
*K*-max (D)	45.20 ± 1.44	45.61 ± 1.36	0.236
TPP (microns)	543.90 ± 22.85	523.40 ± 37.01	0.013
MPE (microns)	8.63 ± 4.35	8.13 ± 2.54	0.556
HOA-RMS	0.08 ± 0.03	0.08 ± 0.03	0.505
Coma-RMS	0.06 ± 0.09	0.04 ± 0.03	0.112

**Table 2:** The safety indices (postoperative CDVA/preoperative CDVA) and efficacy indices (postoperative UDVA/preoperative CDVA) of both groups at 6, 12, and 24 months.

	6 months	12 months	24 months
Safety index			
SMILE	1.11 ± 0.20	1.03 ± 0.23	1.07 ± 0.16
SMILE xtra	1.09 ± 0.19	1.07 ± 0.15	1.03 ± 0.08
*p* value	0.737	0.414	0.369

Efficacy index			
SMILE	1.02 ± 0.15	1.01 ± 0.23	1.05 ± 0.16
SMILE xtra	1.01 ± 0.19	1.05 ± 0.15	1.00 ± 0.00
*p* value	0.842	0.503	0.223

**Table 3:** *K*-avg: Keratometry average; *K*-max: Keratometry maximum; TPP: thinnest point pachymetry; maximum posterior elevation.

	1 month	6 months	12 months	24 months
SMILE: *K*-avg (D)	38.37 ± 1.83	38.42 ± 1.77	38.28 ± 1.74	38.74 ± 1.76
SMILE Xtra: *K*-avg (D)	38.91 ± 1.61	38.33 ± 1.50	38.67 ± 1.92	38.35 ± 1.85
*p* value	0.232	0.837	0.418	0.485
SMILE: *K*-max (D)	41.00 ± 5.87	41.12 ± 4.66	41.27 ± 4.40	41.64 ± 3.53
SMILE Xtra: *K*-max (D)	41.83 ± 6.02	41.21 ± 3.76	41.74 ± 3.56	41.50 ± 2.77
*p* value	0.590	0.340	0.464	0.264
SMILE: TPP (microns)	403.83 ± 35.51	407.94 ± 37.05	414.03 ± 36.28	408.29 ± 38.75
SMILE Xtra: TPP (microns)	399.40 ± 40.82	394.45 ± 38.00	402.16 ± 39.52	402.22 ± 37.18
*p* value	0.664	0.192	0.188	0.600
SMILE: MPE (microns)	12.65 ± 5.85	12.47 ± 7.33	11.13 ± 4.39	10.14 ± 3.85
SMILE Xtra: MPE (microns)	13.22 ± 5.86	14.81 ± 7.02	11.54 ± 5.15	11.00 ± 4.72
*p* value	0.705	0.239	0.747	0.523

**Table 4:** HOA-RMS: high-order aberration root mean square; Coma-RMS: coma root mean square.

	1 month	6 months	12 months	24 months
SMILE: HOA-RMS (microns)	0.17 ± 0.10	0.20 ± 0.20	0.14 ± 0.07	0.13 ± 0.07
SMILE Xtra: HOA-RMS (microns)	0.17 ± 0.09	0.19 ± 0.14	0.12 ± 0.06	0.13 ± 0.07
*p* value	0.835	0.918	0.116	0.736
SMILE: Coma-RMS (microns)	0.12 ± 0.15	0.14 ± 0.34	0.06 ± 0.07	0.12 ± 0.21
SMILE Xtra: Coma-RMS (microns)	0.16 ± 0.18	0.08 ± 0.10	0.06 ± 0.05	0.16 ± 0.25
*p* value	0.440	0.335	0.921	0.589

## Data Availability

The data that support the study are available under directing request.

## References

[1] Binder P. S. (2003). Ectasia after laser in situ keratomileusis. *Journal of Cataract & Refractive Surgery*.

[2] Randleman J. B., Russell B., Ward M. A., Thompson K. P., Stulting R. D. (2003). Risk factors and prognosis for corneal ectasia after LASIK. *Ophthalmology*.

[3] Chen Y. I., Chien K. L., Wang I. J. (2007). An interval-censored model for predicting myopic regression after laser in situ keratomileusis. *Investigative Opthalmology & Visual Science*.

[4] Alió J. L., Muftuoglu O., Ortiz D. (2008). Ten-year follow-up of laser in situ keratomileusis for myopia of up to −10 diopters. *American Journal of Ophthalmology*.

[5] Wollensak G. (2006). Crosslinking treatment of progressive keratoconus: new hope. *Current Opinion in Ophthalmology*.

[6] Hafezi F., Kanellopoulos J., Wiltfang R., Seiler T. (2007). Corneal collagen crosslinking with riboflavin and ultraviolet A to treat induced keratectasia after laser in situ keratomileusis. *Journal of Cataract & Refractive Surgery*.

[7] Spadea L. (2010). Corneal collagen cross-linking with riboflavin and UVA irradiation in pellucid marginal degeneration. *Journal of Refractive Surgery*.

[8] Raiskup-Wolf F., Hoyer A., Spoerl E., Pillunat L. E. (2008). Collagen crosslinking with riboflavin and ultraviolet-A light in keratoconus: long-term results. *Journal of Cataract & Refractive Surgery*.

[9] Kanellopoulos A. J., Binder P. S. (2007). Collagen cross-linking (CCL) with sequential topography-guided PRK: a temporizing alternative for keratoconus to penetrating keratoplasty. *Cornea*.

[10] Kanellopoulos A. J., Pamel G. J. (2013). Review of current indications for combined very high fluence collagen cross-linking and laser in situ keratomileusis surgery. *Indian Journal of Ophthalmology*.

[11] Ivarsen A., Asp S., Hjortdal J. (2014). Safety and complications of more than 1500 small-incision lenticule extraction procedures. *Ophthalmology*.

[12] El-Naggar M. T. (2015). Bilateral ectasia after femtosecond laser-assisted small-incision lenticule extraction. *Journal of Cataract & Refractive Surgery*.

[13] Wang Y., Cui C., Li Z. (2015). Corneal ectasia 6.5 months after small-incision lenticule extraction. *Journal of Cataract & Refractive Surgery*.

[14] Mazzotta C., Traversi C., Mellace P. (2018). Keratoconus progression in patients with allergy and elevated surface matrix metalloproteinase 9 point-of-care test. *Eye and Contact Lens: Science and Clinical Practice*.

[15] Randleman J. B., Woodward M., Lynn M. J., Stulting R. D. (2008). Risk assessment for ectasia after corneal refractive surgery. *Ophthalmology*.

[16] Binder P. S., Trattler W. B. (2010). Evaluation of a risk factor scoring system for corneal ectasia after LASIK in eyes with normal topography. *Journal of Refractive Surgery*.

[17] Osman I. M., Helaly H. A., Abdalla M., Shousha M. A. (2016). Corneal biomechanical changes in eyes with small incision lenticule extraction and laser assisted in situ keratomileusis. *BMC Ophthalmology*.

[18] Ganesh S., Brar S. (2015). Clinical outcomes of small incision lenticule extraction with accelerated cross-linking (ReLEx SMILE Xtra) in patients with thin corneas and borderline topography. *Journal of Ophthalmology*.

[19] Mattila J. S., Holopainen J. M. (2016). Bilateral ectasia after femtosecond laser-assisted small incision lenticule extraction (SMILE). *Journal of Refractive Surgery*.

[20] Roy A. S., Shetty R. (2017). Ectasia after SMILE: correct interpretation of biomechanical hypothesis. *Journal of Refractive Surgery*.

[21] Randleman J. B. (2016). Ectasia after corneal refractive surgery: nothing to SMILE about. *Journal of Refractive Surgery*.

[22] Celik U. H., Alagöz N., Yildirim Y. (2012). Accelerated corneal crosslinking concurrent with laser in situ keratomileusis. *Journal of Cataract & Refractive Surgery*.

[23] Kanellopoulos A. J. (2009). Collagen cross-linking in early keratoconus with riboflavin in a femtosecond laser-created pocket: initial clinical results. *Journal of Refractive Surgery*.

[24] Ng A. L. K., Chan T. C. Y., Cheng G. P. M. (2016). Comparison of the early clinical outcomes between combined small-incision lenticule extraction and collagen cross-linking versus SMILE for myopia. *Journal of Ophthalmology*.

[25] Graue-Hernandez E. O., Pagano G. L., Garcia-De La Rosa G. (2015). Combined small-incision lenticule extraction and intrastromal corneal collagen crosslinking to treat mild keratoconus: long-term follow-up. *Journal of Cataract & Refractive Surgery*.

[26] Zhou Y., Liu M., Zhang T. (2018). *In vivo* confocal laser microscopy of morphologic changes after small incision lenticule extraction with accelerated cross-linking (SMILE Xtra) in patients with thin corneas and high myopia. *Graefe’s Archive for Clinical and Experimental Ophthalmology*.

[27] Tomita M., Mita M., Huseynova T. (2014). Accelerated versus conventional corneal collagen crosslinking. *Journal of Cataract & Refractive Surgery*.

[28] Konstantopoulos A., Liu Y. C., Teo E. P., Nyein C. L., Yam G. H., Mehta J. S. (2019). Corneal stability of LASIK and smile when combined with collagen cross-linking. *Translational Vision Science & Technology*.

[29] Vestergaard A. H., Grauslund J., Ivarsen A. R., Hjortdal J. O. (2014). Efficacy, safety, predictability, contrast sensitivity, and aberrations after femtosecond laser lenticule extraction. *Journal of Cataract & Refractive Surgery*.

[30] Sekundo W., Kunert K. S., Blum M. (2011). Small incision corneal refractive surgery using the small incision lenticule extraction (SMILE) procedure for the correction of myopia and myopic astigmatism: results of a 6 month prospective study. *British Journal of Ophthalmology*.

